# PRRT2, a network stability gene

**DOI:** 10.18632/oncotarget.19506

**Published:** 2017-07-24

**Authors:** Caterina Michetti, Anna Corradi, Fabio Benfenati

**Affiliations:** Center for Synaptic Neuroscience and Technology, Istituto Italiano di Tecnologia, Largo Rosanna Benzi, Genova, Italy and Department of Experimental Medicine, University of Genova, Viale Benedetto, Benzi 10, 16132 Genova, Italy

**Keywords:** paroxysmal disorders, PRRT2 mutations, PRRT2 knockout mouse, synaptic facilitation, network instability

**Figure 1:**
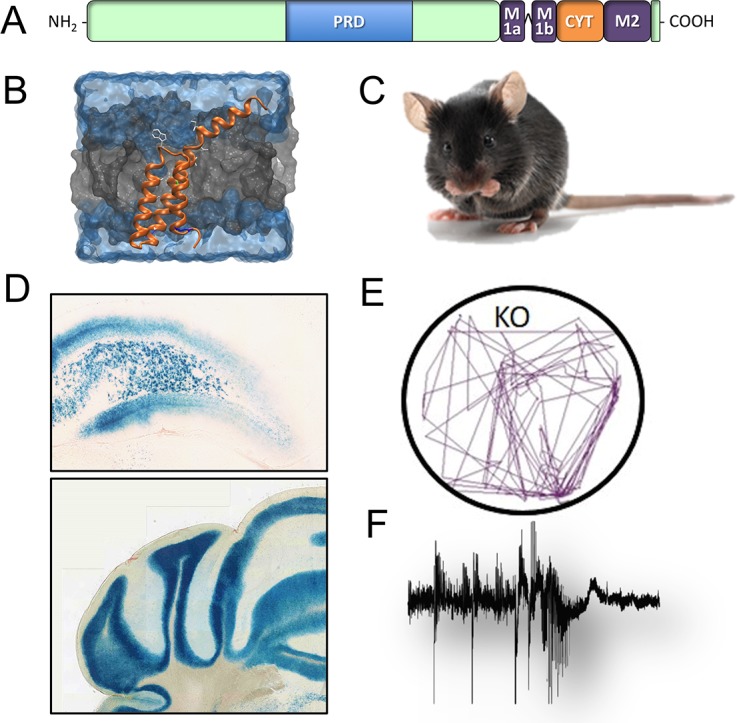
Schematics of PRRT2 domain and 3D structure **A., B.** Regional expression in the brain (β-galactosidase staining; **C., D.**) and paroxysmal phenotype of the PRRT2 KO mouse in response to audiogenic **E.,** or convulsive **F.,** stimuli.

Specific paroxysmal disorders, namely benign infantile epilepsy (BFIE), kinesigenic dyskinesia (PKD), infantile convulsions and choreoathetosis (ICCA) and hemiplegic migraine (HM), are associated with mutations in the gene encoding for PRoline-Rich Transmembrane protein 2 (PRRT2; Figure 1A, 1B). PRRT2 is a neuron-specific protein expressed on neuronal membranes and at synapses, with a prevalent presynaptic location [1, 2]. Several nonsense, missense and frame-shift mutations were identified in the PRRT2 gene, but the vast majority of patients (80%) carry the same frameshift single-nucleotide duplication (c.649dupC) that leads to a premature stop codon and results in a loss-of-function pathogenetic mechanism [3]. Despite the extensive characterization of PRRT2 mutations, no clear evidence for genotype-phenotype correlation exists and the three main diseases (BFIE, PKD and PKD/ICCA) form a continuous spectrum, starting from BFIE in the first months of life and evolving to PKD or PKD/ICCA often in association with HM during adolescence [4-5].

To model the disorders and investigate the underlying neurobiological alterations, we recently characterized a PRRT2 knock out mouse (PRRT2 KO; Figure 1C), carrying a constitutive inactivation of the PRRT2 gene [6]. The promoterless lacZ gene integrated into the PRRT2 locus allowed mapping the PRRT2 regional expression, that is not widespread, but rather concentrated at restricted brain areas. Interestingly, the expression is high in neurons of the lower hindbrain particularly in the cerebellum, a brain area involved in the generation of motor/epileptic phenotype and in which altered synaptic plasticity at the parallel fibers-Purkinje cells synapse was found [6] (Figure 1D). A selective staining was also identified in the cerebral cortex, claustrum and dorsal horns of the spinal cord. Moreover, in the hippocampus, PRRT2 is particularly expressed in the hilus of the dentate gyrus, where mossy cells control the excitability of granule cells, playing a role in preventing hippocampal seizures (Figure 1D). Notably, all the brain regions positive for PRRT2 are involved in processing sensory information, motor disorders and epilepsy, all neurological traits present in patients with PRRT2 mutations.

The PRRT2 KO mouse recapitulates many of the phenotypic features of the human PRRT2-linked disorders, showing abnormal motor behaviors and a motor/epileptic phenotype in response to environmental stimuli [6]. The motor problems are represented by gait abnormalities and a peculiar paroxysmal backwalking appearing early in the postnatal life and persisting in the adult [4]. The motor/epileptic phenotype of PRRT2 KO mice becomes dramatic in response to audiogenic stimuli (Figure 1E), which trigger wild running, backwalking and jumping, and to the administration of convulsants, such as pentylentetrazole (Figure 1F). In both kinds of provocations, however, the seizure propensity was not very severe, consistent with the mild epileptic phenotype of patients bearing PRRT2 mutations [4, 5].

Overall, the PRRT2 KO mouse reproduces the paroxysmal traits described in PRRT2 patients and characterized by sudden and short attacks that usually occur periodically in response to a sensory trigger. The episodic nature of these events suggests that they result from network instability caused by changes in the excitation/inhibition balance. Interestingly, data obtained in primary neurons showed that PRRT2 silencing is associated with alterations of synaptic transmission and short-term plasticity [1]. PRRT2 silencing gives rise to a decreased number of excitatory synapses and a marked impairment of fast synchronous neurotransmitter release. These effects are associated with a decrease in Ca^2+^ sensitivity and release probability, revealing a role for PRRT2 at synaptic level, both during development and in mature synapses. The decrease in release probability is the basis for the highly enhanced facilitation in response to repeated high-frequency stimuli. Such a change was also observed at the parallel fiber-Purkinje cell synapses in the cerebellum of PRRT2 KO mice [6]. It is known that network dynamics, excitability and bursting behavior are highly dependent on the short-term plasticity dynamics of excitatory synapses, rather than on their basal transmission properties, and that enhanced facilitation promotes the propagation of bursting over random spiking events. These observations suggest indicate that the absence of PRRT2, through short-term plasticity changes, impairs the functional stability of neuronal networks, rendering them more susceptible to paroxysmal events triggered by external stimuli. Indeed, paroxysmal dyskinesia patients appear normal between attacks suggesting that, in the absence of specific triggers, homeostatic mechanisms can keep excitability under control.

PRRT2 action at synaptic level is also confirmed by its interaction with several synaptic proteins. The analysis of its exact topology revealed that is a type-2 single-pass membrane protein with a large cytoplasmic N-terminus, a topology similar to the SNARE proteins syntaxin or VAMP/synaptobrevin [7] (Figure 1A, 1B). Indeed, PRRT2 interacts with SNAP-25 and VAMP/synaptobrevin, two components of the SNARE complex responsible for neurotransmitter release, and with the fast Ca^2+^ sensors synaptotagmins (Syts) 1/2. The absence of PRRT2 may cause an uncoupling between the Ca^2+^ sensors and the SNARE complex, providing a molecular basis for the knockdown/knockout phenotype in synaptic transmission and short-term plasticity [1, 6]. These findings suggest that the pathogenetic mechanisms at the basis of paroxysmal disorders may reside in defects of synaptic function. Interestingly, mutations in the PNKD gene were identified in paroxysmal non-kinesigenic dyskinesia, a movement disorder similar to PKD. PNKD is expressed at the synapse and interacts with Rim, a synaptic protein pivotal for synaptic vesicle exocytosis that binds and regulates various types of voltage-gated Ca^2+^ channels [8].

Since previous studies showed that mutations in ion channels are responsible for other paroxysmal dyskinetic phenotypes in the specific mouse models *tottering*, *lethargic* and *moonwalker* [6], it is tempting to speculate that paroxysmal motor symptoms can be generated by dysfunctions in either ion channels or presynaptic proteins regulating short-term synaptic plasticity, leading to network instability/hyperexcitability. In this landscape, the PRRT2 KO mouse emerges as an interesting experimental model to study the molecular basis of paroxysmal disorders and identify novel therapeutic targets to effectively cure these diseases.
